# Women's beliefs about methods and contraceptive discontinuation: Results from a prospective study from Nairobi and Homa Bay counties in Kenya

**DOI:** 10.3389/fgwh.2023.1034634

**Published:** 2023-03-13

**Authors:** Yohannes Dibaba Wado, Martin K. Mutua, George Odwe, Francis Obare, Kazuyo Machiyama, John B. Casterline, John Cleland

**Affiliations:** ^1^ Health and Wellbeing Program, African Population and Health Research Center, APHRC Campus, Nairobi, Kenya; ^2^ Population Council - Kenya, Nairobi, Kenya; ^3^ Faculty of Epidemiology and Population Health, London School of Hygiene and Tropical Medicine, London, United Kingdom; ^4^ Department of Sociology, Ohio State University, Columbus, OH, United States

**Keywords:** discontinuation, method related, beliefs, attributes, Nairobi, Homa Bay

## Abstract

**Background:**

Rates of contraceptive discontinuation are high in many low and middle countries contributing to unmet need for contraception and other adverse reproductive health outcomes. Few studies have investigated how women's beliefs about methods and strength of fertility preferences affect discontinuation rates. This study examines this question using primary data collected in Nairobi and Homa Bay counties in Kenya.

**Methods:**

We used data from two rounds of a longitudinal study of married women ages 15–39 years (2,812 and 2,424 women from Nairobi and Homa Bay respectively at round 1). Information on fertility preferences, past and current contraceptive behavior, and method-related beliefs about six modern contraceptive methods were collected, along with a monthly calendar of contraceptive use between the two interviews. The analysis focused on discontinuation of the two most commonly used methods in both sites, injectables and implants. We carry out competing risk survival analysis to identify which method related beliefs predict discontinuation among women using at the first round.

**Results:**

The percentages of episodes discontinued in the 12 months between the two rounds was 36%, with a higher rate of discontinuation in Homa Bay (43%) than in the Nairobi slums (32%) and higher for injectables than implants. Method related concerns and side effects were the major self-reported reasons for discontinuation in both sites. The competing risk survival analysis showed that the probability of method related discontinuation of implants and injectables was significantly lower among respondents who believed that the methods do not cause serious health problems (SHR = 0.78, 95% CI: 0.62–0.98), do not interfere with regular menses (SHR = 0.76, 95% CI: 0.61–0.95) and do not cause unpleasant side effects (SHR = 0.72, 95% CI 0.56–0.89). By contrast, there were no net effects of three method related beliefs that are commonly cited as obstacles to contraceptive use in African societies: safety for long-term use, ability to have children after stopping the method, and the approval of the husband.

**Conclusion:**

This study is unique in its examination of the effect of method-specific beliefs on subsequent discontinuation for a method-related reason, using a longitudinal design. The single most important result is that concerns about serious health problems, which are largely unjustified and only moderately associated with beliefs about side effects, are a significant influence on discontinuation. The negative results for other beliefs show that the determinants of discontinuation differ from the determinants of method adoption and method choice.

## Background

There is a large body of research on contraceptive discontinuation in low and middle income countries (LMICs), most of it using Demographic and Health Surveys (DHS) data (1–5). This research makes clear that contraceptive discontinuation among women who wish to avoid pregnancy is a common problem. For instance, analysis of DHS data from 19 LMICs showed that, on average, 38% of women discontinue using reversible methods by the 12th month of initiation (3). A similar analysis of DHS data also showed that the 12 month contraceptive discontinuation rate varied from 18% to 63% across the 8 countries studied (1). Although contraceptive discontinuation is very common, family planning programs in LMICs continue to focus on recruiting new users with little attention to current users to promote continuation of use (6).

High rate of contraceptive discontinuation for reasons other than a desire for pregnancy is a public health concern because of its association with negative reproductive health outcomes (2, 6). Contraceptive discontinuation contributes to higher fertility rates, unwanted pregnancies and abortions with adverse effects on maternal, neonatal and child health outcomes (7–9). In their analysis of DHS from 15 countries, Blanc and colleagues found that more than half of unwanted childbearing in those LMICs was the result of births that were preceded by contraceptive failure or discontinuation. They also estimated that, if discontinuation while in need of contraception were eliminated, the total fertility rate (TFR) would be reduced by between 20% and 48% (7). Another study by Ali, Cleland and Shah also indicated that the percentage of accidental pregnancies after contraceptive discontinuation that end in miscarriage, still birth or abortion ranges from 5% to 20% in LMICs (3).

Contraceptive continuation is associated with quality of care (10–12). In their earlier work on contraceptive discontinuation, Ali and Cleland noted that high rates of discontinuation may signal discontent with the method and or family planning service provision and that high failure rates likely indicate inadequate counselling (13). Evidence from DHS also shows that method-related reasons are the pre-dominant cause of contraceptive discontinuation (1, 3, 14, 15). The method-related reasons include experience of side effects, health concerns, access or availability problems, desire for a more effective method and inconvenience of use. Between 2% and 47% of women provide such method-related reasons for discontinuing although still wanting to avoid pregnancy (1, 7). Method related reasons can be addressed through availability of a wide range of contraceptive methods and through adequate counseling that may be supported by educational materials.

These DHS analyses suffer from an important design limitation: the assessment of reasons for discontinuation relies entirely on information from women who have discontinued. Women who do not discontinue may also experience side effects, often the most prevalent reason women report for discontinuation. Relying only on information from women who have discontinued, one cannot assess whether fears and experience of side effects discriminate between those who continue to use and those who terminate use. The same problem applies to other commonly provided reasons such as health concerns, concerns about future infertility, and so forth. The present study, by making use of method specific beliefs measured for all women, corrects this design defect. DHS-type information on the reasons for discontinuing is also available in this study's data.

Past research on correlates of contraceptive discontinuation have considered a range of demographic, socio-economic and health care related factors (4, 5, 14–18). These are factors measured in surveys for continuers and discontinuers alike. Among the factors documented as related to contraceptive discontinuation are women's fertility preferences, age, parity and marital status (5, 16–19). Other studies looked at the role of partner influence and gender related power dynamics on contraceptive adoption, discontinuation and switching, showing higher relative risk of contraceptive discontinuation for women who did not discuss pregnancy avoidance with their partner prior to contraceptive use (20, 21). By comparison, there is an absence of studies that examine how “costs of fertility regulation” (18), broadly defined, influence contraceptive discontinuation. Especially neglected are “non-access costs of fertility regulation” (22, 23) which encompass factors such as concern about health effects (e.g., future capacity to conceive), social acceptability, and partner opposition (12). And while past research has often considered fertility preferences as a determinant, typically this has been operationalized as crude classification of women as wanting to delay the next birth (space) or have no further births (stop), with no attention to strength of motivation to space or stop. This analysis of method-specific contraceptive discontinuation, relying on longitudinal data collected at two sites in Kenya, improves on this past literature by exploiting measurement of method-specific beliefs and fertility preferences (24).

Specifically we hypothesize that users of a specific method who report adverse beliefs about effects of the method, whether based on personal experience (e.g., menstrual disruption or unpleasant side effects) or on scientifically unjustified fears (e.g., serious health problems or permanent infertility) will be more likely to cease use than other users. Further, we hypothesize that women who express particularly strong desires to avoid pregnancy will be less likely to cease use.

## Methods

### Data and study setting

We use data from two rounds of a prospective study on “Improving Measurement of Unintended Pregnancy and Unmet Need for Family Planning”. The information was collected from a cohort of married and cohabiting women between the ages of 15–39 years living in Nairobi Urban Health and Demographic Surveillance System (NUHDSS) and Homa Bay County in Kenya. Women aged 40–49 years were excluded because of their low risk of pregnancy, with many reporting infecundity. The Nairobi study was nested in the NUHDSS implemented by the African Population and Health Research Center (APHRC) where households are visited twice a year to collect data on key socio-demographic and health indicators. The NUDHSS covers two slum settlements of Korogocho and Viwandani in Nairobi. Data collection in Homa Bay County in western Kenya was carried out by the Population Council. The Homa Bay study used a two-stage cluster sampling design to identify households and individual respondents. Detailed information on the study sample size and sampling procedures were described elsewhere in Machiyama et al. 2017 (24), Mumah et al. (2017) (25) and Odwe et al., 2017 (26). Ethical approval was obtained from the African Medical and Research Foundation (AMREF) Ethics and Scientific Review Committee, Kenyatta National Hospital/University of Nairobi Ethics and Research Committee, the African Population and Health Research Center (APHRC) Institutional Review Committee, and Institutional Review Boards at the Population Council's and the London School of Hygiene and Tropical Medicine.

The first round of data collection occurred from August to December 2016 in both Nairobi and Homa Bay and covered 2,812 and 2,424 women from Nairobi and Homa Bay, respectively. In round 1, the surveys collected detailed information on respondent's demographic and socio-economic characteristics, fertility preferences, contraceptive behavior, length of use and intended length of future use, as well as method related beliefs. These same women were re-interviewed a year later from September to December 2017. Those who reported themselves as infecund, sterilized or not in union at round one were excluded from the second round. During round two survey, 2,208 women from Nairobi and 2,083 women from Homa Bay site were successfully re-interviewed with a response rate of 78% and 86%, respectively. The round two survey also covered respondent's reproductive behavior, fertility preferences and contraceptive use as well method related beliefs. This round also inquired about contraceptive discontinuation, namely whether respondent was using same method reported at baseline, and if not reasons for stopping the method.

Analysis of contraceptive discontinuation is based on calendar data collected during the second round survey, which is a month by month history of every birth, pregnancy and episode of contraceptive use a woman had in the 12 months before the second round survey. An episode of contraceptive use is a period of continuous use of a specific method, counted starting from the first month of use until the respondent reports stopping the method, switching to another method or becomes pregnant while using. Accordingly, a switch to another contraceptive method initiates a new episode of use. In any month in which women reported discontinuing contraceptive use, they were asked for the primary reason for stopping the method. The study instrument contained pre-coded reasons including becoming pregnant while using, desire to become pregnant, fear of side effects/health concerns, inconvenience to use, husband's disapproval, access/availability problems, wanting more effective method, infrequent sex, menopause/infecundity, marital dissolution, and other method-related reasons.

### Measures/variables

The analysis is method-specific and carried out for the two most common contraceptive methods in these samples of Kenyan women, namely injectables and implants (if women reported current use of more than one method at round one, we give priority to the more effective method). The observation is episode of contraceptive use at the time of the round 1 interview. Across both sites, there were 2,535 episodes of use of injectables and implants (1,554 injectable, 981 implant). We examine predictors of the discontinuation of these episodes by the round 2 interview, which occurred about twelve months later. The discontinuations include transitioning to a different method (switching) and transitioning to no use. Our interest is discontinuation that women attribute to method-related reasons; these included side effects, health concerns, access/availability problems, desire for a more effective method, inconvenience to use, cost and other unspecified reasons that imply dissatisfaction with the method while still at risk of an unintended pregnancy (11). All other reasons for discontinuation were combined together as “other reasons of discontinuation” in the competing risk survival analysis.

The potential predictors of discontinuation of principal interest are method-specific beliefs about each of the two methods (injectable, implant). Women were asked about eleven method related attributes: their familiarity with the method, knowledge of source and access to the method, perceived use of, and satisfaction with, the method by others in the women's social network, perception of the effectiveness of the method at preventing pregnancy, safety of the method for long-term use, and the likelihoods of the method causing unspecified health problems, unpleasant side effects, menstrual disruption and infertility. Two of the method related attributes, familiarity with method and knowledge of source were dropped from analysis because the analysis is confined to current users. Respondents were also asked whether they believe their husband approves of each method. These method-specific beliefs were collected in both round 1 and round 2; we rely exclusively on round 1 measurement for prediction of discontinuation between rounds 1 and 2. More details on the measurement in this Kenyan data collection are published elsewhere (27, 28). The precise wording and sequence of questions can be found at (http://stepup.popcouncil.org/library/STEPUP_questionnaire_31072016.pdf).

In the analysis, all method-specific beliefs are entered as binary variables. Most items were already simply yes or no response options. A few were reduced to binary form. Specifically, the item “How many of your friends, relatives and neighbors have tried the method?” has response options “most”, “about half”, “few”, “none” and “don’t know”. A binary variable was created by combining women who responded “most”, “about half”, and “few” into one category (coded “1”) and the remaining “none” and “don’t know” to another category (coded as “0”).

Concerned about redundancy of method beliefs, we assessed strength of association between all pairs of beliefs using Cramer's *V*, separately by method and site. Cramer's *V* is a measure of association between two nominal variables, giving values between 0 and 1. Higher values indicate greater association. For most the value of *V* was less than 0.2, indicating weak associations. The principal exception was the association between belief in unpleasant side effects and menstrual disturbance, for which the values of *V* were in the range of 0.41 and 0.54, indicating moderate associations. A particular concern was the possibility of strong associations of beliefs in serious health problems and beliefs about bleeding and other side effects. *V* values for the bleeding-health problems associations were low, between 0.19 and 0.29, but slightly higher for other side effects, in the range of 0.32 and 0.42. In short, this assessment revealed no associations so high (Cramer's *V* > 0.50) that necessitated the exclusion of one or more method beliefs. Hence we include all beliefs in the analysis.

The second explanatory variable distinctive to this research was “strength of fertility preferences” referring to women's motivation to delay or avoid getting pregnant. The survey collected detailed information on the fertility desires of women and their spouses. The future fertility preferences question asks women whether they would like to have (a/another) child, or would prefer not to have any (more) children. For those who want a child in the future, additional questions were asked to find out how long they want to wait from the date of the interview before the birth of another child. The fertility preferences variable was initially recoded into five categories; want a child soon or within 2 years, want a child in 2–4 years, want a child after 5 years, want no more child, and undecided/not sure or missing categories.

Beyond this standard DHS questioning, the survey assessed the strength of women's motivation to avoid pregnancy by asking the following question; “How important is it to you to avoid becoming pregnant now: Would you say very important, somewhat important, or not at all important?”. Additional questions were asked to assess women's feelings, asking if she becomes pregnant in the next few weeks whether she would be worried about telling her husband/partner; whether she would be worried about how she could afford to raise her children properly with an extra child; and whether she would be concerned or not about the effect on her own health. In the analysis, these variables on the importance of avoiding pregnancy and women's feelings were recoded to binary form to create a variable of “strength of motivation”. Response options were coded as 1 for “worried” and 0 for “not worried or unsure” and then summed to give an overall score which was then classified into binary form indicating strong vs. weak motivation based on their responses to the four survey questions. We then combined the two variables (fertility preferences and strength of motivation) and created a composite variable of strength of fertility preferences with five categories: want no more/want to wait 5 years or longer with strong motivation; want no more/want to wait 5 years or longer with weak motivation; want to wait 2–4 years with strong motivation; want to wait 2–4 years with weak motivation; and want a child soon (within two years) or undecided.

Background variables serving as confounding are age of the respondent, education, and study site (Nairobi vs. Homa Bay). Age was recoded into two categories as 15–24 and 25–39 years. Respondent's highest level of education was recoded into three categories; no schooling or some primary education, completed primary, and secondary or higher than secondary education.

### Statistical approach

Analysis focused on discontinuation of injectables and implants**.** Episodes of use of other methods were too few to sustain analysis. The unit of analysis is episode of continuous use of a specific method. The distinction between method-related and non-method-related reasons for discontinuation is fundamental to this analysis. Our interest is correlates of method-related discontinuation. Episodes discontinued for other reasons (e.g., wanting to become pregnant) are included in the regression analysis, which employs a competing risks approach with two competing risks: discontinuation for method-related and for non-method-related reasons.

In a first stage of the analysis, we examine the stated reasons for the discontinuation of episodes between round 1 and round 2. This analysis is confined to women who discontinued, and it is a bivariate analysis with no attention to covariates.

In the second stage of the analysis, we model the predictors of discontinuation, separately for injectables and implants. For this portion of the analysis, we adopt a formal survival analysis methodology because the contraceptive episodes are characterized by both left-censoring and right-censoring. The former, also known as “delayed entry”, occurs because episodes were already in process at the time of the round 1 survey; the latter occurs because some episodes were still in process at the time of the round 2 survey. Survival analysis is designed to accommodate both types of censoring when calculating relative rates of discontinuation (29). We applied a competing risks survival analysis (method-related vs. non-method-related reasons for discontinuation). This is a special type of survival analysis that aims to correctly estimate marginal probability of an event in the presence of competing events (29). Traditional survival analyses are not designed to accommodate the competing nature of multiple causes of the same event and therefore tend to produce inaccurate estimates when analyzing the marginal probability for cause-specific events (29, 30). Thus, Cumulative Incidence Function (CIF) from competing risk survival analysis is proposed to address this issue by estimating the marginal probability of a certain event as a function of its cause-specific probability and overall survival probability. We employ the Fine and Gray approach as implemented in Stata procedure “stcrreg” (29, 30). Incidence functions and sub-hazard ratios (and corresponding confidence intervals) can be calculated for each competing risk. In this analysis, we calculate these only for method-related discontinuation.

The survival analysis is conducted with the data pooled across the two sites (Nairobi and Homa Bay). We have carried out site-specific analysis and confirmed that the main findings do not differ between sites. Moreover, the site-specific analysis suffers from weak statistical power. We have also adjusted standard errors for clustering for the Homa Bay data where the survey involved multi-stage cluster sampling.

## Results

### Characteristics of the study population

The characteristics of the study participants interviewed at the first round of the survey are presented in Table 1. The majority (71.8%) of the respondents were aged 25–39 years, with respondents from rural (Homa Bay) site relatively younger than the urban (Nairobi) site (65.8% vs. 76.9%). Less than one-third of the respondents (32%) had secondary or above education, with relatively higher proportion of respondents from the urban site having attained secondary or higher education than the rural site. The mean number of living children was 2.8, ranging from 2.4 in Nairobi to 3.4 in Homa Bay. With regards to strength of fertility preference, over half of women in both sites wanted to have no more children or delay the next child for five or more years. In Nairobi about half of these women were classified as having a strong motivation, whereas in Homa Bay only about one-third were classified as strongly motivated. About 20% in both sites reported a desire to postpone the next birth for between two to four years, and this spacing preference was more likely to be held strongly in Homa Bay than in Nairobi (Table 1).

**Table 1:** Percent distribution of married or cohabiting women aged 15–39 years at round 1 in the two sites by background characteristics, Kenya 2016.

Variables	Nairobi		Homa Bay		Total	
	%	*n*	%	*n*	%	*n*
**Current age**						
15–24	23.1	649	34.2	830	28.2	1479
25–39	76.9	2163	65.8	1594	71.8	3757
**Highest level of education**						
No education/some primary	21.1	592	44.8	1086	32.1	1678
Primary complete	39.4	1107	32.7	792	36.3	1899
Secondary and above	39.6	1113	22.5	546	31.7	1659
**Fertility Preference/strength of motivation**						
Want soon or within 2 years, undecided	23.4	613	17.1	415	19.6	1028
Want a child in 2–4 years with weak motivation	15.3	400	11.9	288	14.1	688
Want a child in 2–4 years with strong motivation	5.5	143	12.1	292	8.3	435
Want no more/want to wait 5 + years with-weak motivation	26.8	702	37.5	910	30.8	1,612
Want no more/want to wait 5 + years-strong motivation	29.0	758	21.4	519	24.4	1,277
Others*/missing	7.0	196	-	-	3.7	196
**Number of living children**						
None	3.5	98	3.3	81	3.4	179
1	27.0	759	13.2	320	20.6	1079
2	32.2	905	20.3	491	26.7	1396
3	19.7	554	21.1	512	20.4	1066
4+	17.6	496	42.1	1020	29.0	1516
Mean	2.4	2,733	3.4	2,357	2.8	5090
**Contraceptive use at round 1**						
Not using	19.2	540	25.5	618	22.1	1158
Using	74.1	2084	64.5	1565	69.6	3647
Pregnant	6.7	188	10.0	243	8.2	431
**Contraceptive methods used** **						
Pill	8.1	229	2.5	61	5.5	290
Injectable	32.3	907	26.7	647	29.7	1554
Implant	19.5	549	17.8	432	18.7	981
Sterilization	0.9	24	2.7	66	1.7	90
Male condom	1.7	47	8.6	208	4.9	255
Rhythm	6.1	170	3.4	83	4.8	253
Other traditional***	3.1	107	2.2	53	3.1	160
**Total**	**100**	**2812**	**100**	**2424**	**100**	**5236**

* Others include women who said they can’t get pregnant, sterilized and missing cases.

** *N* includes women who reported use of a method at round 1.

*** Includes traditional methods such as withdrawal and Lactational Amenorrhea Method (LAM).

### Contraceptive use and discontinuation

Contraceptive use is high in this sample of currently married women in the prime reproductive ages (under age 40): nearly 70% of the respondents reported using a method of contraception at the time of the first round survey, with higher proportion of users in Nairobi (74.1%) than in Homa Bay (64.5%). Injectables (29.7%) and implants (18.7%) were the most commonly used methods in both Nairobi and Homa Bay. At the time of the round 1 survey, the median duration of use among current users of injectables and implants was only 3 months for each method. Among other methods, the use of rhythm was more common in Nairobi and male condom in Homa Bay (Table 1).

The percentage of episodes discontinued in the months between the two rounds was 36%, with a higher percentage of discontinuation in Homa Bay (43%) than in the Nairobi slums (32%). In both sites, higher percentage of discontinuation were reported for injectables than implants. The discontinuation of injectables was 44% in Homa Bay compared to 31% in Nairobi, and the discontinuation of implants was 22% in Nairobi compared to 16% in Homa Bay. By contrast the proportion of discontinued episodes that were followed by switching to other methods was higher in Nairobi (19.0%) than in Homa Bay (17.4%) and for injectables (17.1% and 15.2% in Nairobi and Homa Bay, respectively) than implants (14.3% and 6.4% in Nairobi and Homa Bay, respectively). Other than these two methods, discontinuation rates were particularly high for short term and traditional methods (pills, condom, and rhythm). For instance, more than half of users of pills (69%) and male condom (55%) discontinued between the round 1 and round 2 surveys in Homa Bay (Tables 2, 3).

**Table 2:** Percent distribution of discontinuations of contraceptive methods in the 12 months preceding the survey by main reason stated for discontinuation, according to specific method, Homa Bay, Kenya 2017.

Method	Implants	Injectables	Pills	Condoms	Rhythm	Other	Total
Method failure	8.8	10.6	11.6	8.7	28.2	16.0	11.6
Desire to become pregnant	13.8	12.5	16.3	10.3	23.1	4.0	12.4
Other fertility related reasons	1.3	6.0	14.0	19.1	5.1	2.0	8.3
Side effects/health concerns	51.3	46.8	20.9	2.4	0.0	2.0	29.5
Wanted more effective method	2.5	2.3	9.3	28.6	28.2	42.0	13.3
Other method related	5.0	11.3	23.3	21.4	7.7	10.0	13.1
Other/DK	17.5	10.6	4.7	9.5	7.7	24.0	11.8
Total (discontinuation rate for any reason)	15.7	43.9	68.8	54.9	42.8	72.9	43.3
Switching to another method	6.4	15.2	36.7	27.7	21.1	26.8	17.4
*N* (Episodes)	508	821	100	329	105	91	1954

**Table 3:** Percent distribution of discontinuations of contraceptive methods in the 12 months preceding the survey by main reason stated for discontinuation, according to specific method, Nairobi, Kenya 2017.

Method	Implants	Injectables	Pills	Condoms	Rhythm	Other	Total
Method failure	4.8	5.1	13.6	11.1	12.9	3.2	7.3
Desire to become pregnant	18.6	20.8	18.9	16.7	29.0	17.7	20.4
Other fertility related reasons	2.4	4.2	6.1	5.6	4.8	1.6	4.1
Side effects/health concerns	52.4	48.2	32.6	0.0	0.0	22.6	38.4
Wanted more effective method	0.0	7.4	10.6	27.8	32.3	19.4	10.4
Other method related	4.0	2.2	9.1	22.2	3.2	11.3	5.2
Other/DK	17.7	12.1	9.1	16.7	17.7	24.2	14.2
Total (discontinuation for any reason)	22.0	31.2	48.0	48.2	34.3	29.1	32.2
Switched to another method	14.3	17.1	25.0	36.3	20.9	22.4	19.0
*N* (Episodes)	555	1035	345	47	209	224	2415

The major stated reasons for discontinuation varied by contraceptive method and site. In both the urban and rural sites, more than half of women who discontinued implants stopped due to experience of side effects and health concerns (Tables 2, 3). Similarly, about 48% of women who discontinued injectables from Nairobi and 47% of those from Homa Bay stopped due to experience of side effects and health concerns. The desire to become pregnant was the second main reason for stopping injectables and implants in both Homa Bay and Nairobi. A considerable proportion of implant users mentioned other reasons for discontinuation, which included desire to take a break from the implant, stopping after the method expired, and infrequent sex due to husband's absence. The reasons for discontinuation of other contraceptives were also similar. For instance, about a third of women who stopped pills in Nairobi discontinued due to experience of side effects and health concerns. The desire for more children and the search for more effective methods also ranked among the major causes of discontinuing methods like male condom, rhythm and other traditional methods (Tables 2, 3).

### Method-related beliefs

The Kenyan data contain women's beliefs about a variety of attributes of each of the two methods examined here (injectable and implant) using chi-square test. The data from both sites show near consensus that both methods are easy to obtain, effective in preventing pregnancy, and easy to use. Women's perception of the health effects of methods was rather mixed. The majority of women (>70%) from both sites reported that implants and injectables are unlikely to cause serious health problems and do not cause infertility. However, less than 50% of respondents from both sites reported that the methods do not interfere with regular menses, do not cause unpleasant side effects and are safe for long-term use. In both sites, more than 90% of respondents reported that they have a friend, a relative or neighbor who have used the two methods. However, between 35%–43% said that they have friends/relatives/neighbors who have tried and are not satisfied with the methods. In both sites, husband approval was higher for injectables than implants (Table 4).

**Table 4:** Beliefs about the methods among current users of injectables and implants at round one.

Method attribute	Nairobi			Homa Bay		
	Injectables	Implants	*p*-value	Injectables	Implants	*p*-value
	%	%		%	%	
**Convenience/effectiveness**						
Easy to obtain	97.7	92.0	0.846	91.8	86.1	<0.001
Effective at preventing pregnancy	92.9	93.9	0.292	92.0	92.4	0.822
Easy to use	93.5	87.4	0.778	92.3	81.9	0.003
**Health effects or concerns**						
Unlikely to cause serious health problems	85.6	80.4	<0.001	66.3	58.8	<0.001
Does not interfere with regular menses	24.2	16.7	<0.001	33.2	24.2	<0.001
Does not causes unpleasant side effects	45.3	34.5	<0.001	44.1	33.1	<0.001
Safe for long-term use	44.9	36.3	<0.001	50.2	42.9	<0.001
Does not cause infertility	72.9	79.9	<0.001	80.1	80.4	0.552
**Social networks**						
Husband approves of method	91.1	84.1	0.006	77.1	74.5	<0.001
Have a friend/ relative/ neighbor who used the method	95.7	92.5	<0.001	93.1	91.1	<0.001
Friends/relatives/neighbors are satisfied with method	65.1	57.2	0.013	58.5	57.1	0.431

### Results of competing risk survival analysis

Table 5 shows the sub-hazard ratios (SHR) and 95% confidence intervals from the competing risk survival analyses predicting women's likelihood of discontinuing implants and injectables due to method related reasons. The unadjusted analysis shows that the sub-hazard ratio of discontinuing injectables and implants did not vary significantly by women's age, education and fertility preferences but vary by study site and contraceptive method. After adjusting for confounders, the probability of discontinuing was 42% higher for injectable users (SHR = 1.42, 95% CI: 1.01–1.99) compared to implant users. The probability of discontinuation was also slightly higher in Homa Bay (SHR = 1.25, 95% CI: 1.02–1.53) than in Nairobi.

**Table 5:** Unadjusted (crude) and adjusted competing risk survival models predicting women's probability of discontinuing implants or injectables during 12-month follow-up, Nairobi and Homa Bay.

	Unadjusted (crude) model SHR [95%CI]	Adjusted model^a^ SHR [95%CI]
**Age Group (RC = 15–24 years)**		
25–39 years	0.89 (0.72–1.08)	0.90 (0.76–1.07)
**Site (RC = Nairobi)**		
Homa Bay	1.17 (1.00–1.36)*	1.25 (1.02–1.53)*
**Educational attainment** (Ref: no education/incomplete primary)		
Completed primary	0.98 (0.78–1.23)	1.01 (0.80–1.27)
Secondary and above	0.99 (0.77–1.28)	1.05 (0.80–1.38)
**Method** (RC = Implant)		
Injectable	1.50 (1.12–2.01)**	1.42 (1.01–1.99)*
**Fertility preference** (Ref = Want no more/ want to wait 5 + years with strong motivation)		
Want no more/want to wait 5 + years with weak motivation	1.04 (0.82–1.33)	1.02 (0.80–1. 29)
Want to wait 2–4 years with weak motivation	1.24 (0.99–1.55)	1. 08 (0.80–1.47)
Want to wait 2–4 years with strong motivation	1.21 (0.89–1.63)	1.18 (0.95–1.46)
Want soon/want within 2 years/undecided	1.29 (1.023–1.63)*	1.20 (0.94–1.53)
**Method related beliefs**		
Methods easy to obtain	1.08 (0.77–1.51)	1.07 (0.74–1.56)
Effective at preventing pregnancy	1.34 (0.70–2.57)	1.22 (0.98–1.54^)^
Easy to use methods	1.08 (0.77–1.46)	1.22 (0.98–1.54)
Unlikely to cause serious health problems	0.72 (0.61–0.85)**	0.79 (0.64–0.99)*
Unlikely to interfere with regular menses	0.74 (0.65–0.85)**	0.89 (0. 74–1.01)*
Unlikely to cause unpleasant side effects	0.68 (0.60–0.76)**	0.79 (0.68–0.91)**
Safe to use for long without stopping	0.89 (0.74–1.08)	1.05 (0.83–1.32)
A woman may not have children after stopping method	0.94 (0.75–1.67)	0.96 (0.78–1.20)
Social network tried and satisfied	0.84 (0.73–0.97)*	0.82 (0.70–0.97)*
Husband/partner approves method	0.91 (0.80–1.03)	0.99 (0.81–1.21)
**Number of Observations**	2008	2008

SHR, sub-hazard ratio.

^a^ Adjusted for Methods related beliefs, fertility preference, Method, Education attainment, site and age of the woman; RC, reference category.

* *p* < .05.

** *p* < .01. ±*p* < 0.10.

After adjusting for the effects of socio-demographic factors (age, education and study site) and fertility preferences, predictive power varies among the method-related beliefs. The probability of discontinuation was significantly lower among women who believed that the methods are unlikely to cause unpleasant side effects (SHR = 0.79, 95% CI: 0.686–0.91) and women who believed the method is unlikely to cause serious health problems (SHR = 0.79, 95% CI: 0.64–0.99). In addition, the probability of discontinuation was also significantly lower among women whose social networks used and approved the methods (SHR = 0.82, 95% CI 0.70–0.97). By contrast, and strikingly, there are no unadjusted or adjusted effects of three method-related beliefs; safety for long-term use without stopping, fear of infertility, and perceived approval of the husband. As documented in Table 4, substantial fractions of the respondents held such beliefs at the round 1 interview, yet these beliefs do not predict discontinuation.

The fertility preference variable, which reflects both desire to avoid pregnancy (space or stop) and strength of this desire, was not significantly associated with discontinuation. The bivariate analysis (column “Crude SHR”) also shows that discontinuing due to method related-reasons did not vary significantly based on women's fertility preferences or the strength of motivation to space or limit pregnancy.

## Discussion

The study examines the effects of women's beliefs about methods and strength of fertility preferences on the discontinuation of two of the most commonly used contraceptive methods in Nairobi and Homa Bay counties in Kenya. The study confirms findings from other surveys such as the DHS and Performance Monitoring for Action (PMA) that contraceptive use is a common practice among married women in Kenya, with more than two-thirds of married women from Nairobi site and about two-thirds from the rural Homa Bay county using some form of family planning at the time of the baseline survey in 2016.

However, adverse and inaccurate beliefs about the major contraceptive methods are common and contraceptive discontinuation remains a major problem with about one in three users discontinuing a method during the twelve-month follow up period. Overall, the two sites had higher contraceptive prevalence and higher discontinuation rate than the national average (31).

An earlier analysis from the same project showed that current users of a method have more positive beliefs about the method than past or never users (27). Nevertheless, substantial minorities of women who were current users at baseline believed that their method might cause serious health problems and infertility. Even higher proportions, over half in Nairobi and about half in Homa Bay, considered their method unsafe for long term use without taking a break. These results underscore the fact that many women in Kenya use a method despite concerns about its safety. Beliefs about serious health problems, infertility and safety for long-term use are largely unjustified and not based on personal experience. Conversely, beliefs about menstrual disruption and side effects, associated with injectables and implants, are justified and presumably are based on personal experience. It is unsurprising, therefore, that the majority of users in both sites reported negative beliefs on both methods.

The competing risk survival analysis showed that the sub-hazard of discontinuation from method-related causes was higher among users of injectable contraception compared to implant users. The relationship between the types of contraceptive methods used and contraceptive discontinuation is relatively well documented, with higher probability of discontinuation consistently reported for traditional and short-term methods (including injectables) than long-term and more effective methods such as implants (1, 5, 15, 32). Moreover, the 2014 Kenya DHS indicates that higher percentages of implant users were informed about side effects or problems of methods used and what to do if side effects are experienced than injectable users (31).

Although some previous studies from Kenya and elsewhere reported association between women's age and contraceptive discontinuation (15, 17), neither women's age nor educational status were significantly associated with method related discontinuation in this study. However, the sub-hazards of discontinuation due to method-related reasons varied between the two sites with marginally significant higher probability of discontinuation in Homa Bay than Nairobi. This may reflect differences in the quality of information and counselling services provided on contraceptive methods and for implants, accessible removal services between the rural (Homa Bay) and urban (Nairobi) sites.

To our knowledge, this is the first study in LMICs to examine the effects of women's beliefs about methods on method-specific discontinuation for reasons that imply dissatisfaction with the method. Bearing in mind that our analytic sample was current users at baseline, five of these beliefs were almost certainly based on personal experience. Three of these concerned perceptions that the method was easy to obtain, easy to use, and effective at preventing pregnancy. Large majorities of women in both sites had positive beliefs on these topics. None were significantly associated with discontinuation after adjustment. The other two beliefs, that the method does not interfere with regular menses and does not cause unpleasant side effects, were predominantly negative and both were significantly associated with discontinuation.

The importance for discontinuation of bleeding-related and other side effects identified in this study is consistent with a large body of evidence from other studies in Kenya and elsewhere, particularly from self-reported reasons for stopping a method (1, 3, 15). One study found that an additional day of menstrual bleeding was significantly associated with a 2%–4% increase in discontinuation, depending on method type (33). It is also consistent with earlier analyses from the same project in Homa Bay and Nairobi. Among past users, beliefs about bleeding and other side effects were strongly associated with overall satisfaction with the method and satisfied past use was the strongest predictor of method-specific adoption between rounds 1 and 2 (26, 34, 35). Further valuable insights come from an additional round of data collection in Homa Bay but not in Nairobi (36). Over half of current users of injectables and implants reported an effect on regular menses, most commonly irregular bleeding, and about one-third regarded the bleeding side effect as very serious.

Three further health-related beliefs concerning serious health problems, permanent fertility impairment and safety for long-term use, were measured. Unlike the side effects discussed above, these are not based on personal experience and are largely unjustified. As hypothesized, beliefs about serious, unspecified health problems, were marginally associated with discontinuation, after adjusting for other beliefs. In most published work on self-reported reasons for discontinuation, side effects are combined with health concerns, with the implication that they are difficult to distinguish from each other. However, we found that beliefs about side effects, whether related to bleeding or other symptoms, were only moderately associated with the belief that the method might cause serious health problems. Our results show that they can and should be considered separately and that they exert independent influences on method-related discontinuation.

Contrary to expectations, neither of the other two beliefs was associated with discontinuation, even before adjustment for covariates, despite, in the case of infertility, a substantial literature that shows such fears are highly prevalent (37, 38). One possible reason for the negative result in this study is that current users, self-evidently, have overcome fears about infertility, perhaps because the need to avoid pregnancy is regarded as more important or because they want no more children, in which case concern about future fertility impairment is no longer relevant. Some evidence to support these speculations comes from the earlier analysis of method adoption between round one and two (34). In Nairobi, where most women wanted to space births but not limit family size, negative beliefs about infertility influenced method choice but not in Homa Bay where limitation was the more common desire. In the case of safety for long-term use, for which negative beliefs were common, the explanation may be that the length of follow-up was too short to detect an influence.

The final two beliefs to be assessed were the influence of social networks and husband's approval of the method. A large body of evidence shows that fertility and family planning behavior is affected by the experiences and attitudes of friends, neighbors and relatives, though little is known about such social influences on discontinuation(20, 25, 39). In this study, over 90% of current users knew members of their social network who had used the same method and between 57% and 65% of these members, depending on site and method, were perceived to be satisfied. After adjustment, the effect of satisfied use by social networks was significantly related to discontinuation. It thus appears that social influences extend beyond willingness to adopt contraception and choice of method to persistence of use.

As with social networks, a large body of evidence shows that the attitude of the husband to childbearing and contraception exerts a major influence on a couple's reproductive behaviour. Evidence on the husband's influence on continuation of use is sparse though a recent study in Uganda showed that the relative risk of discontinuation doubled when women had not discussed pregnancy avoidance with their partner prior to adoption (20). In this study, the majority of users reported that their husband approved of the method, though approval was higher in Nairobi than Homa Bay. Approval had no association with discontinuation. Clearly, the minority of women who adopt a method despite perceived spousal disapproval are sufficiently empowered to disregard partners' views or perhaps use the method in a clandestine manner. It should be noted, however, that husband's approval was a strong predictor of adoption of a method between rounds one and two (34).

Our study did not show significant association between reported future fertility preferences or with strength of motivation to space or limit childbearing and method-related discontinuation of implants and injectables. This finding may reflect limitations in our construction of this variable. However, analysis of baseline fertility preferences, presented at an IUSSP workshop on Methodologies for Measuring Pregnancy Intention and Unintended Pregnancy and Birth, held in May 2021, shows that they are predictive of pregnancy between round one and two. In both sites, women wanting no more children or to postpone the next birth for five or more years were less likely to have a pregnancy than those wanting a child sooner. In addition, concern about the financial implications of having another child added to predictive power. One obvious explanation for this apparent discrepancy is that women with stronger motivation to avoid childbirth for a long period of time are more likely to switch to an alternative method after discontinuation. This is a priority for future analysis. An alternative explanation is that they are more likely to terminate any pregnancy. Such behavior is unlikely to be reported.

This study is unique in its measurement of the effect of women's beliefs about methods on subsequent discontinuation of use, with a longitudinal design. However, it has limitations. While the measurement of method-specific beliefs is a special feature of the Kenyan data, it should be noted that we are unable to specify whether beliefs about the two methods existed at the time of initial adoption or developed while the method was being used. Stability of beliefs over time is a further concern. In addition, while we used standard and validated tools overall, the construction of one composite variable “strength of fertility preferences” may not adequately represent the intended construct. There are also limitations associated with longitudinal data collection. First, a small proportion of women reported using different methods retrospectively (during the second round survey) than methods reported during the first round. A small number of women also reported a baseline pregnancy retrospectively while the round one survey indicated otherwise. These and other inconsistences in the data were resolved by excluding such cases from the analysis or by changing their baseline exposure status for those who reported pregnant retrospectively for the reason that women in the first trimester may not know they were pregnant. Although the recall period is relatively short in this study, evidence from DHS surveys show that under reporting of prior method use, particularly of short-term and traditional methods, is common (1, 5). A further minor limitation stems from the fact that women aged 40 or more years were omitted from the study.

## Conclusion

Although contraceptive use is high in the two communities, contraceptive discontinuation is also high, potentially exposing women to unintended pregnancies. More than one-third of episodes of contraceptive use were discontinued during the twelve-month follow up period. Method-related factors accounted for more than half of the reasons for discontinuation of implants and injectables. Our results support the large body of evidence that side effects are major causes of discontinuation. In our study, the association with discontinuation of both types of side effects were approximately equal. An important contribution is to show that concerns about serious health problems can be distinguished from side effects and that these concerns have an independent influence on discontinuation. This finding calls for information campaigns and individual counselling to address erroneous beliefs. A further important insight from comparing these findings with prior analysis of adoption is that the influences on length of use are very different from the influences on propensity to adopt a method. Strong evidence exists that fear of infertility, perception of husband's approval, and nature of future childbearing desires are determinants of contraceptive adoption and method choice. Yet none of these factors were associated with discontinuation.

## Data Availability

The raw data supporting the conclusions of this article will be made available by the authors, without undue reservation, upon request.
